# Mechanisms of ferroptosis and the relationship between ferroptosis and ER stress after JEV and HSV infection

**DOI:** 10.3389/fmicb.2024.1415417

**Published:** 2024-09-11

**Authors:** Rui Zhou, Kexin Wei, Xinyu Li, Beibei Yan, Lin Li

**Affiliations:** ^1^Shanxi Bethune Hospital, Shanxi Academy of Medical Sciences, Third Hospital of Shanxi Medical University, Tongji Shanxi Hospital, Taiyuan, China; ^2^First Hospital of Shanxi Medical University, Shanxi Medical University, Taiyuan, China

**Keywords:** JEV, HSV, Ferroptosis, LPO, ROS, ER stress

## Abstract

Ferroptosis is a novel form of programmed cell death, which is different from apoptosis, pyroptosis and autophagy in morphology and biochemistry. Ferroptosis is characterized by condensed mitochondrial membrane densities, vanished of mitochondria crista and outer membrane rupture in morphology, and the accumulation of intracellular iron, lipid peroxidation (LPO), decrease of GSH and inhibition of GPX4 in biochemistry. Japanese encephalitis virus (JEV) and Herpes simplex virus (HSV) are both common neurotropic viruses that can cause neurological disorders, such as severe encephalitis. JEV and HSV have been demonstrated to be able to induce ferroptosis. This process is closely related to the inhibition of the GSH-GPX4 system, ACSL4 phosphorylation, and Nrf2 ubiquitination. In this review, we summarized the mechanisms by which JEV and HSV induced ferroptosis in the current study. In addition, we found a strong relationship between endoplasmic reticulum (ER) stress and ferroptosis, and we therefore speculated that sustained ER stress might be a prerequisite for ferroptosis in JEV and HSV-induced diseases.

## Introduction

Japanese encephalitis virus (JEV) is a neurotropic pathogen characterized by high infectivity and rapid diffusion. Its infection leads to extensive neuronal damage and inflammation within the central nervous system (CNS), which causes lethal encephalitis (Yang et al., 2024). Clinical symptoms caused by JEV are characterized by neurological symptoms such as meningitis, encephalitis, and myelitis, as well as flaccid paralysis and seizures. In severe cases, lesions may even be observed in the thalamus, basal ganglia, midbrain, hippocampus, and cerebral cortex (McMillan et al., 2023).The pathologic hallmarks of JEV-infected patients are neuron loss and microglia activation (Liao et al., 1998). Herpes simplex viruses (HSVs) are one of the most prevalent neurotropic viruses, including HSV type 1 and type 2 (HSV-1 and HSV-2) (James et al., 2020), which establish lifelong latent infection in keratinized epithelial surface, and sensory neurons and dorsal root ganglia of the peripheral nervous system (He and Han, 2023; Ren et al., 2023).Diseases caused by HSV include cold sores, genital herpes, herpes stromal keratitis (HSK), eczema herpeticum, disseminated disease in the neonate, meningitis and herpes simplex encephalitis (HSE) (Zhu and Viejo-Borbolla, 2021). In addition, several neurodegenerative diseases have also shown a link to HSV infection (Zhu and Viejo-Borbolla, 2021). Two neuroinvasive viruses, JEV and HSV, both cause severe encephalitis, and the current studies found a strong correlation between symptoms after JEV and HSV infection and ferroptosis (Xu et al., 2023; Zhu et al., 2024). Ferroptosis is a new non-apoptotic form of programmed cell death discovered in recent years, which is usually accompanied by a large amount of iron accumulation and lipid peroxidation (LPO) during cell death. Ferroptosis inducers can directly or indirectly affect glutathione peroxidase through different pathways, resulting in a decrease in antioxidant capacity and accumulation of lipid reactive oxygen species (ROS) in cells, ultimately leading to oxidative cell death (Li et al., 2020). This review summarized and compared the mechanisms of ferroptosis induced by JEV and HSV, and speculated that endoplasmic reticulum (ER) stress might be a prerequisite for ferroptosis in JEV and HSV-induced diseases.

### JEV and HSV

JEV is a member of the Flavivirus genus of the Flaviviridae family, which is highly prevalent and the major cause of inducing viral encephalitis in the Asian region (Zhu et al., 2023). JEV is transmitted to humans through the bites of Culex mosquitoes, particularly Culex tritaeniorhynchus. JEVs are reproduced in animal hosts such as pigs and poultry which act as viral reservoirs. In recent years, studies on the mechanism of JEV-induced cell death have revealed its association with ferroptosis and endoplasmic reticulum stress (ER stress) (Zhu et al., 2024).

HSV is widely distributed in the population with a high infection rate, and its main route of transmission is close contact and sexual contact (Rathbun and Szpara, 2021). HSV causes chronic infection in humans that are characterized by periodic episodes of mucosal shedding and ulcerative disease (Rathbun and Szpara, 2021), which can cause a variety of diseases, such as cold sores, genital herpes, meningitis, and HSE (Zhu and Viejo-Borbolla, 2021). Besides human beings, HSV can infect a variety of animals such as rabbits, mice and other experimental animals, which shows a very wide host range. HSV can proliferate and replicate in a variety of cells leading to cytopathic effects (Esteves et al., 2018; Kutle et al., 2023). HSV infection initially occurs in epithelial cells of the orolabial and genital mucosa as well as in the skin and cornea (Sun et al., 2024). After replication in epithelial cells, HSVs reach and enter neurons and establish lifelong latency in the ganglia of the peripheral nervous system (PNS) (Sun et al., 2024). There are two serotypes of HSV, HSV-1 and HSV-2, with similar genome structure, about 50% homology of nucleic acid sequence and about 83% homology of protein coding region. They show many biological similarities, but can be distinguished by sequence analysis or restriction enzyme profiling (Arvin et al., 2007). The transmission routes of the HSV-1 and HSV-2 are different. HSV-1 is mainly transmitted through close contact, while HSV-2 is mainly transmitted through sexual contact or neonatal genital tract infection, so that results in different clinical manifestations of the disease. Clinically, HSV-1 infection is usually shown in oral niches and HSV-2 infection is often observed in genital niches (Rathbun and Szpara, 2021).

Current studies have demonstrated that the ferroptosis inhibitor Liproxstain-1 (Lip-1) inhibited neuronal death and LPO caused by JEV infection (Zhu et al., 2024). In addition, some inhibitors of ferroptosis or proteasome were found to block HSV-1-induced nuclear factor E2-related factor 2 (Nrf2) degradation, thereby effectively alleviating nerve damage and inflammation in HSV-1 infected mice (Xu et al., 2023). This applied that ferroptosis and ER stress might play a key role in the pathological processes caused by JEV and HSV infections. The study of these mechanisms provides new insights into how viruses affect host cells, which may provide potential targets for the development of new therapeutic strategies.

**Figure 2:**
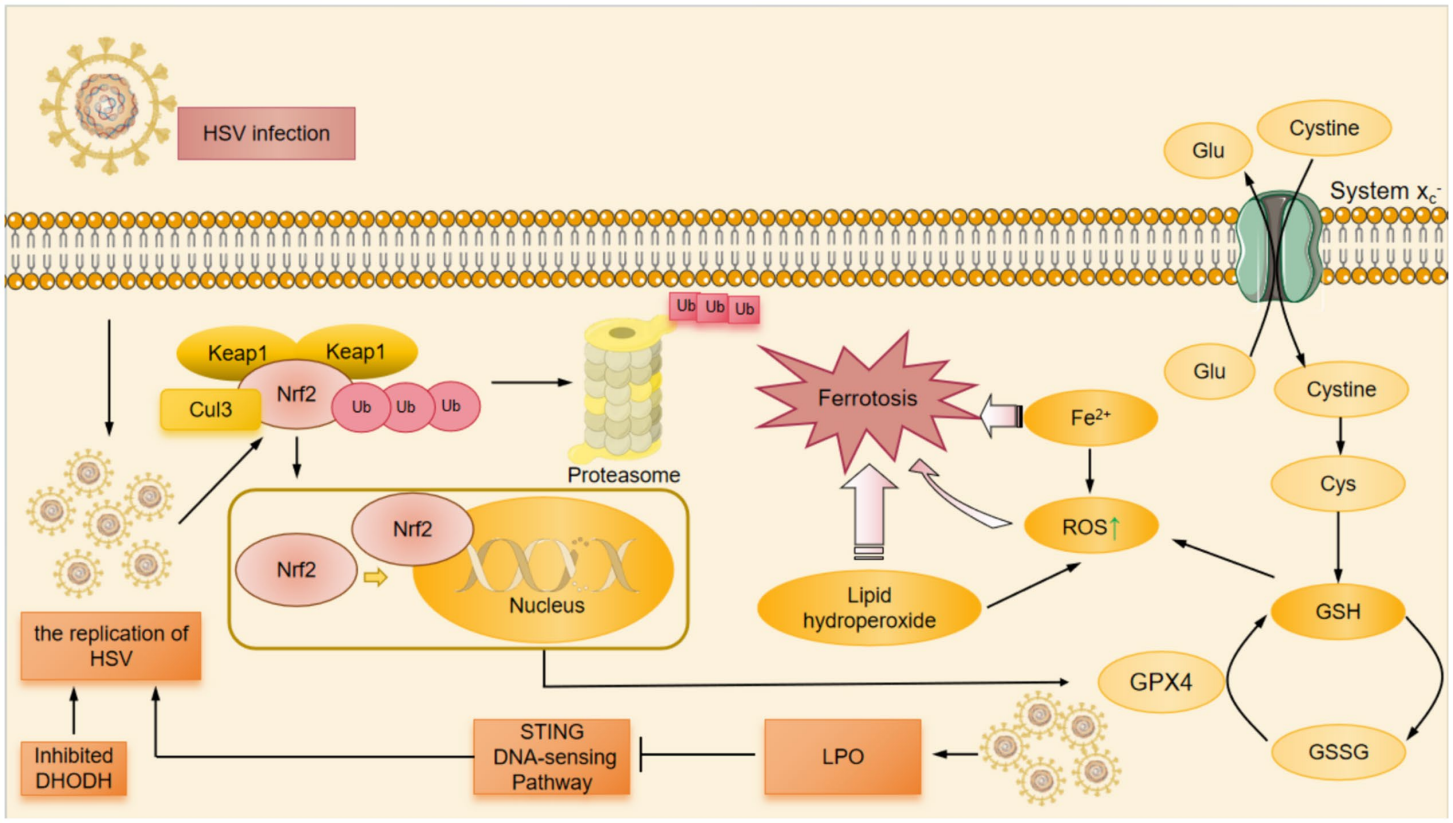
The mechanism of HSV-induced ferroptosis. HSV infection induced LPO which down-regulated the STING DNA-sensing pathway. The down-regulation of STING DNA-sensing pathway inhibited the innate cell damage pathway and suppressed the innate antiviral immune response against HSV, thereby promoting HSV replication. At the same time, DHODH had the ability to inhibit HSV replication. When DHODH was inhibited, HSV replication in the body was enhanced. HSV infection led to the degradation of Nrf2 through the ubiquitin proteasome pathway. Keap1 was an adaptor component of the Cul3-based ubiquitin E3 ligase that enhanced the ubiquitylation of Nrf2, which promoted the degradation of Nrf2 in proteinsome. The degradation of Nrf2 resulted in a decrease in the amount of Nrf2 that was translocated to the nucleus for expression of antioxidant-associated genes, which suppressed cellular GPX4 expression. Cysteine was a substrate for the synthesis of GSH, and the System Xc- facilitated the exchange of cysteine and Glu. Among other things, HSV infection inhibitd cellular GPX4 expression and significantly downregulated GSH levels.The down-regulation of GSH resulted in the ROS accumulation. In HSV-infected cells, the above pathways induced Fe^2+^ overload, ROS accumulation, GSH depletion, and ultimately ferroptosis. Cul3, Cullin3; DHODH, dihydroorotate dehydrogenase; GPX4, glutathione peroxidase 4; GSH, glutathione; GSSG, glutathione disulfide; Keap1, Kelch-like ECH-associated protein 1; Nrf2, nuclear factor erythroid derived 2-like 2; ROS, reactive oxygen species; STING, stimulator of interferon genes; Ub, ubiquitylation.

### Ferroptosis

Ferroptosis is a novel form of programmed cell death, which is different from apoptosis, pyroptosis and autophagy in morphology and biochemistry. Ferroptosis is characterized by condensed mitochondrial membrane densities, vanished of mitochondria crista and outer membrane rupture in morphology, and the accumulation of intracellular iron, LPO, decrease of glutathione (GSH) and inhibition of glutathione peroxidase 4 (GPX4) in biochemistry (Li and Li, 2020).

#### JEV induced ferroptosis

JEV-induced neuroinflammation and neuronal injury have been demonstrated to be associated with ferroptosis. JEV infection significantly promoted LPO in SH-SY5Y cells and mouse primary neurons in a time-dependent manner (Zhu et al., 2024). After the employ of the ferroptosis inhibitor Lip-1, a significant decrease of LPO was found in JEV-infected SH-SY5Y cells, and viral titers and inflammation were reduced in JEV-infected mice ultimately improving the survival rate of infected mice (Zhu et al., 2024). Neuronal ferroptosis can be induced by JEV infection through two pathways. On the one hand, JEV infection destroyed the antioxidant system in neuronal cells by inhibiting the GSH-GPX4 signaling axis. On the other hand, JEV infection promoted LPO mediated by yes-associated protein 1 (YAP1)/long-chain acyl- CoA (coenzyme A) synthetase 4 (ACSL4) in neurons (Zhu et al., 2024).

The GSH-GPX4 signaling axis is an important intracellular antioxidant pathway, which is essential for maintaining the redox homeostasis in cells. GPX4 is a key antioxidant enzyme responsible for reducing harmful LPO to harmless lipid alcohols so as to protect cells against oxidative damage. JEV infection inhibited GPX4 expression and significantly down-regulated the level of GSH in SH-SY5Y cells (Zhu et al., 2024). Meanwhile, JEV induced ferroptosis was distinctly suppressed with the overexpression of GPX4 that GPX4 converted GSH to GSSH to reduce LPO in SH-SY5Y cells (Li and Li, 2020; Zhu et al., 2024). These studies demonstrated that JEV could damage the antioxidant system in neuronal cells by inhibiting the GSH-GPX4 signaling axis, which in turn led to the occurrence of ferroptosis.

**Figure 1:**
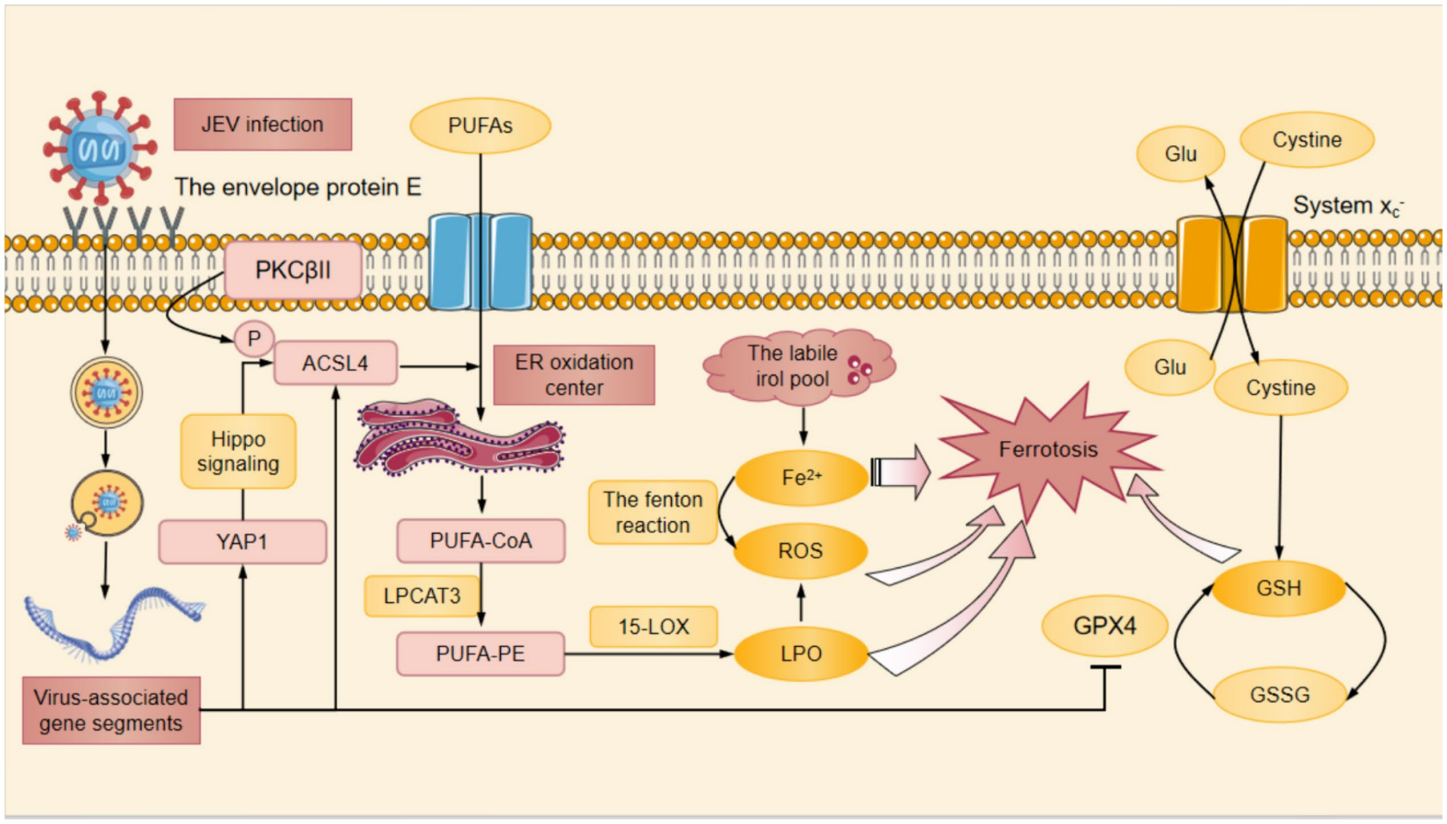
The mechanism of JEV-induced ferroptosis. At first, the envelope protein E of JEV bound to cellular receptors and mediates endocytosis, subsequently the viral membrane fused with the endosomal membrane to invasive host cells. After entering the host cell, JEV was processed to form viral-associated gene segments. On the one hand, JEV infection promoted the expression of YAP1, which mediated the upregulation of ACSL4 through the Hippo signaling pathway. On the other hand, PKCβII phosphorylated the Thr328 site of ACSL4 directly activating ACSL4 and promoting the biosynthesis of PUFAs. JEV infection also promoted the phosphorylation of ACSL4 and contributed to the binding of PUFA to CoA in the ER oxidation center to form PUFA-CoA. With the help of LPCAT3, PUFA-CoA was converted to PUFA-PE. PUFA-PE was then oxidized by 15-LOX to produce the lipid hydrogen peroxide. Subsequently, Fe^2+^ was released from the labile iron pool, resulting in the fenton reaction to produce ROS. Additionally, the System Xc^−^ promoted the exchange of cysteine and Glu. Once cysteine entered the cytoplasm, it was catalyzed to produce GSH from cysteine. Among these, JEV infection inhibited the expression of GPX4 in cells and significantly downregulated GSH levels. Decreased GSH, accumulation of Fe^2+^ and ROS, and increased LPO finally caused ferroptosis after JEV infection. ACSL4, Acyl-CoA synthetase long-chain family member 4; CoA, coenzyme A; ER, endoplasmic reticulum; Glu, glutamate; GPX4, glutathione peroxidase 4; GSH, glutathione; LPO, lipid peroxidation; LPCAT3, lysophosphatidylcholine acyltransferase 3; PKCβII, protein kinase C beta type II; PUFAs, polyunsaturated fatty acids; PUFA-CoA, polyunsaturated fatty acid-coenzyme A; PUFA-PE, polyunsaturated fatty acid-phosphatidylethanolamine; ROS, reactive oxygen species; YAP1, yes-associated protein 1; 15-LOX, 15-Lipoxygenase.

ACSL4 is one of the important signaling molecules that regulate ferroptosis (Yuan et al., 2016), and its increased expression in neurons results in neuronal LPO and ferroptosis after JEV infection. ACSL4 was phosphorylated directly at Thr328 site, which promoted the biosynthesis of polyunsaturated fatty acid (PUFA) lipids (Zhao et al., 2024). PUFA bound to coenzyme A (CoA) in the ER oxidation center to form PUFA-CoA that was further esterified to PUFA-phosphatidylethanolamine (PUFA-PE) with the assistance of lysophosphatidylcholine acyltrans ferase 3 (LPCAT3) (Jia et al., 2023). Then PUFA-PE was oxidized by 15-lipoxygenase (15-LOX) to produce lipid hydroperoxide that contributed to iron depletion (Jia et al., 2023). Subsequently Fe^2+^ was released from the labile iron pool leading to the production of ROS such as HO by the fenton reaction (Jia et al., 2023). In addition, JEV infection promoted the expression of YAP1 that was the upstream of ACSL4 in SH-SY5Y cells (Cottini et al., 2014). YAP1 mediated the upregulation of ACSL4 through TEA domain transcription factor 1 (Tead1)/ TEA domain transcription factor 4 (Tead4)-related Hippo signaling, thus increasing LPO levels and causing ferroptosis in skeletal muscle cells (Yang et al., 2020; He et al., 2022) (Figure 1).

#### HSV induced ferroptosis

HSV has been demonstrated to be able to induce ferroptosis. Fe^2+^ overload, ROS accumulation, GSH depletion, LPO, and mitochondrion shrinkage were observed in HSV-1-infected cultured human astrocytes, microglia and murine brains, which are hallmarks of ferroptosis (Xu et al., 2023).The ways of ferroptosis induced by HSV are related to Nrf2, GSH-GPX4 and dihydroorotate dehydrogenase-dihydroubiquione (DHODH-CoQH2) (Mao et al., 2021; Xie et al., 2022).

HSV-1 infection enhanced the ubiquitination and degradation of Nrf2 mediated by Kelch-like ECH-related protein 1 (Keap1) so as to inhibit the expression of antioxidative genes, which disturbed cellular redox homeostasis and promoted ferroptosis (Uruno and Yamamoto, 2023; Xu et al., 2023). Studies showed that the decrease of Nrf2 down-regulated GPX4, depleted GSH and caused the ferroptosis in HSV-1 infected cells (Xu et al., 2023). Nrf2 is a transcription factor from the cap-n-collar family that harbors a unique basic leucine zipper motif and plays as a master regulator of homeostatic responses (Uruno and Yamamoto, 2023). Keap1 is an adaptor component of Cullin 3 (Cul3)-based ubiquitin E3 ligase that enhances the ubiquitylation of Nrf2, which promotes the degradation of Nrf2 in proteinsome (Uruno and Yamamoto, 2023; Xu et al., 2023). Nrf2 depletion exacerbates symptoms and enhances oxidative damage and inflammation in the CNS of mouse models of neurodegenerative diseases. Nrf2 has recently been found to be a negative regulator of interferon-driven HSV-2 antiviral response. Gunderstofte’s study demonstrated that genetic activation of Nrf2 increased the infectiability and the replication of HSV-2 in Keap1 (−/−) mouse embryonic fibroblasts (Gunderstofte et al., 2019).

Additionally, GSH-GPX4 and DHODH-CoQH2 also participated in the ferroptosis induced by HSV. Stimulator-of-interferon genes (STING) is vital to innate immune responses against microbial infection and tumors and for sensing cytosolic DNA (Jia et al., 2020). GPX4 deficiency enhanced cellular LPO and specifically attenuated the STING DNA-sensing pathway, thereby inhibiting innate antiviral immune responses against HSV-1 and promoting HSV-1 replication *in vivo* (Jia et al., 2020) These suggested that GPX4 facilitated STING activation by maintaining redox homeostasis of lipids (Jia et al., 2020). Although the decrease of Nrf2 down-regulated GPX4 expression Nrf2 did not impair STING mRNA and protein expression levels in murine cells (Gunderstofte et al., 2019).

In addition, dihydroorotate dehydrogenase (DHODH) was found to have the ability to inhibit the replication of HSV-1 and HSV-2 (Luganini et al., 2021). When DHODH was inhibited, the replication of HSV-1 and HSV-2 was also enhanced in the body (Luganini et al., 2021) (Figure 2).

### ER stress and Ferroptosis

JEV and HSV infections trigger rapid aggregation of viral proteins in the ER lumen and stimulate massive ER membrane rearrangements, leading to ER stress and activation of the unfolded protein response (UPR). ER stress itself is a self-protection mechanism rapidly initiated by cells under specific environmental stress, while apoptosis, autophagy and ferroptosis will be induced when ER homeostasis cannot be maintained due to continuous and severe stress (Szegezdi et al., 2006). UPR is a process of removing excess, unfolded, misfolded proteins from the cell. UPR is associated with three ER transmembrane receptors: protein kinase R-like endoplasmic reticulum kinase (PERK), activating transcription factor 6 (ATF6) and inositol-requiring kinase 1 (lRE1) in mammalian cells (Szegezdi et al., 2006).

After ER stress, PERK-eIF2α-ATF4-CHOP signaling pathway is activated, which leads to the occurrence of apoptosis (Rozpedek et al., 2016; Lee et al., 2018). ER stress can increase the activity of PERK that phosphorylates eIF2α at Ser51 site which is involved in protein translation. Phosphorylated eIF2α inhibits the translation of messenger RNA into protein, thereby reducing protein translation during ER stress (Harding et al., 1999). When eukaryotic initiation factor 2α (eIF2α) is phosphorylated, only ATF4 and the C/EBP homologous protein (CHOP) are selectively translated. The increased expression of CHOP leads to the down-regulation of B-cell lymphoma-2 (Bcl-2) gene expression, an anti-apoptotic protein, and increases the burden of new proteins in the ER, thereby promoting ER stress and cell death (Feldman et al., 2005). At the same time, the increased expression of CHOP up-regulates the expression of pro-apoptotic protein BH3 domain genes (such as BIM) and destroys the redox homeostasis, which rapidly causes cell apoptosis (McCullough et al., 2001).CHOP, on the other hand, promotes the expression of ER REDOX protein 1α (ERO1α) gene. The oxidative activity of ERO1 is directly related to the production of H_2_O_2_, promoting the formation of a high oxidative environment. High concentrations of ROS in the ER lumen may also subsequently activate the ER calcium release channel type 1 inositol 1,4,5-trisphosphate receptor (IP3R1), leading to the leakage of calcium from the ER lumen into the cytoplasm. Subsequently, the calcium-sensing kinase calcium/calmodulin-dependent protein kinase II (CaMKII) is activated to induce various apoptotic signaling pathways. Activation of the CHOP-ERO1α-IP3R1-CaMKII pathway induces membrane-bound nicotinamide adenine dinucleotide phosphate oxidase (NADPH oxidase) subunit 2 (NOX2), resulting in ROS production (Tabas and Ron, 2011). ROS can also lead to the activation of CaMKII through a positive feedback loop, thereby promoting the expression of DNA damage-inducible transcript 3 (DDIT3), the gene encoding the transcription factor CHOP (Vandewynckel et al., 2013).

Under persistent ER stress, the blockage of PERK-mediated global translation leads to the activation of IRE1/XBP1 (x-box binding protein 1) and ATF6 pathways, which in turn promotes the expression of various ER chaperones and ERAD mechanical components to enhance protein folding capacity and degrade irreversibly damaged proteins (Sharma et al., 2021). ROS production is exacerbated by reduced protein synthesis, an increase of protein folding capacity, and removal of misfolded proteins, resulting in the accumulation of iron, all of which provide the necessary conditions for ferroptosis (Han et al., 2013). In addition, activation of XBP1 and ATF6 pathways induced by ER stress significantly enhances JEV-induced cell death (Sharma et al., 2017).

The regulated IRE1-dependent decay (RIDD) pathway is part of the cell response to ER stress, and it works by altering the activity of IRE1α that is activated when unfolded or misfolded proteins accumulate in the ER (Bashir et al., 2021). An increase of IRE1α expression enhances cell sensitivity to ferroptosis (Jiang et al., 2024). In the context of ER stress, the RIDD pathway plays a cytoprotective role, while IRE1α promotes the production of XBP1s, which in turn promotes the expression of genes beneficial to ER stress recovery (Shishova et al., 2022). Inhibition of the RNase activity of IRE1 by drugs reduces viral protein levels and viral titers produced by JEV-infected mouse neuroblastoma cells (Neuro2a), which suggests that the activation of the RIDD pathway may have a direct benefit on JEV replication (Bhattacharyya et al., 2014). However, when ER stress persists, RIDD pathway may induce apoptosis (Mukherjee et al., 2017).

The present study has shown that JEV infection activates all three sensors of PERK/eIF2α, IRE1/XBP1, and ATF6 signal-mediated UPR (Wang et al., 2019; Sharma et al., 2021). JEV can interact with PERK via its Nonstructural protein 4B (NS4B) to trigger the PERK/eIF2α/ATF4/CHOP apoptotic pathway and activate neuronal apoptosis (Kumar et al., 2020). During direct neuronal infection, JEV can induce UPR by stimulating ER assessed by CHOP, Mitogen-activated protein kinase p38 (MAPK p38), and RIDD pathways, which leads to neuronal apoptosis (Aktepe and Mackenzie, 2018; Barrows et al., 2018).

In the early stages of HSV-1 replication, only ATF6 was activated by proteolysis and its downstream targets, namely ER partners GRP78 and P94, were not upregulated, which enhanced the replication of HSV in cells and improved viral load (Burnett et al., 2012). In later stages of HSV-1 replication, ATF4 and CHOP were activated, which in turn caused various forms of cell death to release the virus (Burnett et al., 2012). It had been shown that protein kinase R (PKR) was activated in the later stages of HSV-1 infection enhancing phosphorylation of eIF-2α (Chou et al., 1995) and inducing phosphorylation of endoplasmic reticulum resident kinase (pERK) (Cheng et al., 2005), thereby inhibiting protein synthesis (Poppers et al., 2000). In addition, IRE1 was also activated during HSV-1 infection (Su et al., 2017).

In summary, it is not difficult to find that continuous ER stress can accumulate necessary material conditions for the occurrence of ferroptosis such as ROS and Fe^2+^ through UPR and can enhance the sensitivity of cells to ferroptosis by the increased expression of IRE1α in the process of JEV and HSV infections. These mechanisms make ferroptosis more likely to occur in JEV and HSV infected cells. When ferroptosis occurs, the function of GPX4-GSH and other antioxidant systems is impaired resulting in the accumulation of LPO. At the same time, the cell death signal generated by CHOP and other pathways promote cell death, and cause ER stress again, which accumulates conditions for a new round of ferroptosis. We therefore speculate that sustained ER stress may be a prerequisite for ferroptosis in JEV and HSV-induced diseases.

## Discussion

Although this review focuses on the mechanisms of ferroptosis induced by JEV and HSV, the occurrence of ferroptosis is prevalent in many neurotropic virus-induced diseases. For example, following HIV (Human immunodeficiency virus) infection, the HIV-1 Tat protein led to an increased expression of ACSL4 and thus ferroptosis. In addition, the occurrence of LPO, a decrease in GPX4, and mitochondrial damage were also observed after HIV infection. These changes could activate microglia and released pro-inflammatory cytokines, which in turn triggered neuroinflammation and nerve damage (Kannan et al., 2023). Epstein–Barr virus (EBV) is another neurotropic virus. EBV infection promoted PUFA synthesis resulting in ferroptosis (Gao et al., 2023), which might later cause damage in CNS. These examples suggested that ferroptosis might play an important role in neurodegenerative pathogenesis by promoting oxidative stress, exacerbating mitochondrial dysfunction, activating neuroinflammatory responses, and engaging in aberrant protein aggregation ultimately leading to neuronal damage and death. Thus the study of ferroptosis will provide new perspectives and potential targets for the treatment of neurodegenerative diseases.

The exploration of mechanisms of ferroptosis induced by JEV and HSV infection will provide physicians with new ideas for diagnosis and treatment, such as the uses of ferroptosis inhibitors. JEV infection induced neuronal ferroptosis by inhibiting the function of the GSH/GPX4-mediated antioxidant system and by promoting YAP1/ ACSL4-mediated LPO. Using the ferroptosis inhibitor Lip-1, viral titres and inflammatory responses were reduced in the brains of JEV-infected mice and increased survival rate of infected mice (Zhu et al., 2024). Inhibition of Nrf2 expression by a ferroptosis inhibitor (Fer-1) or proteasome inhibitor (MG132) effectively inhibited HSV-1 encephalitis. Meanwhile, PTGS2 inhibitor (Indomethacin) effectively inhibited the expression of multiple inflammatory factors caused by HSV-1 (Xu et al., 2023). Studies on the mechanisms of ferroptosis induced by JEV and HSV will guide patients’ medication profiles and provide physicians with new ideas for diagnosis and treatment. At present, there are several main categories of ferroptosis inhibitors, and we described their types and mechanisms in the following table (Table 1).

**Table 1:** Inhibitors of ferroptosis and their mechanisms.

Types	Inhibitors of ferroptosis	Mechanisms
Iron chelator	Deferoxamine	They induced iron depletion by reducing Fe^2+^ in the dynamic iron pool, inhibited the Fenton reaction, interfered with iron-dependent LPO, reduced lipid reactive oxygen species, and ultimately inhibited ferroptosis. Bruzzese et al. (2023)
	Deferiprone	
	Deferasirox	
	Curcumin	
	Thermodynamics	
Antioxidant	Ferrostain-1	It inhibited ferroptosis by inhibiting LPO accumulation as free radical trapping antioxidants (Tang et al., 2021; Luo et al., 2023)
	Liproxstain-1	It cleared ROS and activated of Nrf2 pathway, and restorated of GPX4 levels (Cao et al., 2021)
	Trolox	It inhibited LPO. Czubak et al. (2017)
	XJB-5-131	It targeted mitochondria to clear ROS. Xun et al. (2022)
Lipoxygenase (LOX) inhibitors	Zileuton	It inhibited 5-LOX (Kahnt et al., 2021)
	AA861	
	PD146176	It inhibited 5-LOX (Da-Costa-Rocha and Prieto, 2021)
	huangcunia (undistilled Chinese alcohol, made of fermented grains)	It inhibited 12/15-LOX
ACSL4 inhibitor	Troglitazone	They inhibited ACSL4 function, blocked PUFA activation and phospholipidation processes, and reduced the generation of LPO raw materials (Singh et al., 2024)
	Rosiglitazone	
	Pioglitazone	
Nitrogen oxide	TEMPO	They blocked fenton reaction and inhibited hydroxyl radical generation (Shi et al., 2017)
	PHOXNO	
Selenium supplementation	Selenium	They supplemented the GPX4 abundance and enhanced lipid peroxide scavenging capacity (Kumar et al., 2011; Friedmann Angeli and Conrad, 2018)
	Methylselenocysteine	

To date, although investigators have developed a variety of ferroptosis inhibitors, many of them are poorly active or have poor pharmacokinetic properties, which limits their further clinical application. Therefore, the development of novel and high-quality ferroptosis inhibitors and their use in clinical treatment of related diseases will be one of the future directions in the field of ferroptosis.

## Author’s note

Infection of neurotropic viruses (JEV and HSV) caused ferroptosis by the accumulation of Fe^2+^, production of ROS, and LPO, which was regulated by GSH-GPX4, ACSL4 and Nrf2.

It was speculated that ER stress might be a prerequisite for ferroptosis in JEV and HSV-induced diseases.
